# Chemical Fingerprints of Honey Fermented by Conventional and Non-Conventional Yeasts

**DOI:** 10.3390/molecules30112319

**Published:** 2025-05-26

**Authors:** Dorota Kregiel, Urszula Dziekonska-Kubczak, Karolina Czarnecka-Chrebelska, Katarzyna Pielech-Przybylska

**Affiliations:** 1 Department of Environmental Biotechnology, Lodz University of Technology, Wolczanska 171/173, 90-530 Lodz, Poland; 2 Institute of Fermentation Technology and Microbiology, Lodz University of Technology, Wolczanska 171/173, 90-530 Lodz, Poland; urszula.dziekonska-kubczak@p.lodz.pl (U.D.-K.); katarzyna.pielech-przybylska@p.lodz.pl (K.P.-P.); 3 Department of Biomedicine and Genetics, Medical University of Lodz, Mazowiecka 5, 92-215 Lodz, Poland; karolina.czarnecka@umed.lodz.pl

**Keywords:** honey wort, fermentation, *M. pulcherrima*, co-cultures, metabolic profiles

## Abstract

Previous studies have shown the positive effects of non-conventional *Metschnikowia* spp. yeasts in mixed cultures with *Saccharomyces cerevisiae* on the properties of fruit wines. In this study, we investigated the effects of using conventional *S. cerevisiae* and non-conventional *Metschnikowia pulcherrima* yeasts as starter cultures in controlled mixed fermentations of honey wort. Other non-conventional yeasts were also tested for comparison, including *Wickerhamomyces anomalus*, *Dekkera*/*Bretannomyces bruxellensis*, and *Wickerhamomyces anomalus*. We evaluated the tolerance of the tested yeasts to high sugar content and analyzed the metabolic profiles of both monocultures and mixed systems. The *M. pulcherrima* strain showed the highest tolerance to 30% *w*/*v* glucose. The chemical complexity of fermented honey was improved using *M. pulcherrima* in co-starters with *S. cerevisiae.* The fermented honey samples were characterized by lower ethanol content, higher glycerol level, and rich volatilomes containing higher levels of both esters (ethyl acetate, 3-methylbutyl acetate, 2-methylpropyl acetate) and aliphatic alcohols (2-methylpropan-1-ol, 3-methylbutan-1-ol, and 2-methylbutan-1-ol). Similar characteristics were obtained using mixed populations of four strains: *S. cerevisiae*, *M. pulcherrima*, *D. bruxellensis*, and *W. anomalus*.

## 1. Introduction

Non-conventional yeasts have until recently been regarded as undesirable microorganisms in winemaking, responsible for cloudiness, incomplete fermentation, low alcohol formation, and the production of undesired aromas in final products [1]. However, non-*Saccharomyces* yeasts, including *Metschnikowia* sp., are now being marketed as active yeast preparates [2,3,4]. Currently, there are more than 80 validated *Metschnikowia* species. The pigmented *Metschnikowia* strains are the most frequently used for winemaking [5], in particular, *M. pulcherrima* and *M. fructicola*, belonging to the *M. pulcherrima* clade [3]. *Metschnikowia* spp. are generally recommended in winemaking for their contribution to improving aromatic complexity. This is due to their enzymatic properties (e.g., β-D-glucosidase, cysteine, β-lyase, esterase), which lead to interesting wine flavors (esters, higher alcohols) [6,7,8]. Numerous studies have described the positive effects of *Metschnikowia* strains in mixed cultures with the classical wine yeast *S. cerevisiae* [9,10,11,12]. Lately, studies have also shown that the *M. pulcherrima* clade can be used to ferment apple and chokeberry wines with improved aromatic complexity, especially in the case of apple wines using the *Metschnikowia* spp. as co-starters with *S. cerevisiae* Tokay [13]. The use of the *M. pulcherrima* clade for the bioprotection of fruit musts as an alternative to using sulphites has also been investigated [14].

Various mechanisms have been proposed to explain the antimicrobial antagonism exhibited by the *M. pulcherrima* clade. One proposed mechanism is related to their ability to produce pulcherrimin. The cells secrete pulcherriminic acid into the environment, which forms an insoluble pulcherrimin chelate with ferric ions. The lack of iron caused by the bonding of ferric ions inhibits the growth of many undesirable microorganisms. As a result, the *M. pulcherrima* clade shows strong biocontrol activity against yeast genera detrimental in enology (e.g., *Brettanomyces*/*Dekkera*, *Wickerhamomyces*, *Hanseniaspora*, *Pichia*), as well as filamentous fungi (e.g., *Penicillium*, *Aspergillus* and *Fusarium*). However, it has no or only a slight negative impact on conventional wine yeasts [7,13,15].

The promising results of the use of *Metschnikowia* species in enology encouraged further research into their application in the production of mead. Mead is a traditional alcoholic beverage obtained by fermenting honey wort. References to mead have been found dating back 3000 years. The origins of the drink can be traced back to African countries. It was later produced throughout the Mediterranean basin and Europe, playing an essential role in early ancient civilizations [16,17]. Traditionally, honey can be diluted with different proportions of water or juice, such as 1:0.5, 1:1, 1:2, or 1:3. In traditional mead, small amounts of fruits, spices, and herbs may also be added, but their incorporation should not overpower the unique flavor and aroma of the mead [16]. Mead contains 8 to 18% *v*/*v* of ethanol. It has not only a long history but also an expanding global market [18].

The yeasts used in the production of mead are usually conventional *S. cerevisiae*, similar to those used in wine or champagne production. These yeasts are able to ferment sugars, such as glucose and fructose, resulting in the formation of ethanol and carbon dioxide. However, given the high sugar levels in honey and wine must and the low nitrogen concentrations in honey, it was hypothesized that these strains may not be the most suitable for mead production [19]. In previous studies conducted by our team, some *M. pulcherrima* strains showed high osmotic tolerance (30% *w*/*v*) [20], which suggested potential for honey fermentation and mead production.

In the present study, we evaluate the chemical properties of Polish honey fermented by yeast monocultures and co-cultures of conventional and non-conventional yeasts. Particular attention was paid to the compatibility of the strains *S. cerevisiae* and *M. pulcherrima* in the presence of other non-conventional yeasts during the first stages of fermentation. The quality of the fermented honey samples was evaluated on the basis of their chemical characteristics, particularly their aroma profiles.

## 2. Results and Discussion

### 2.1. Yeast Growth at Different Concentrations of Glucose

The results of the growth of yeast monocultures after 7-day incubation in YPD medium are presented in Table 1. The tested yeasts were able to grow in the media with the highest concentration of glucose (30% *w*/*v*), but their growth was very diverse.

With increasing glucose concentrations, *M. pulcherrima* showed higher osmotolerance, with a notable increase at 30% glucose concentration in comparison to other tested strains. The increase was statistically significant compared to *D. bruxellensis* at 20% and 30% glucose (*p* = 0.019; *p* = 0.013, respectively, K-W test).

The statistical analysis showed that as the glucose concentration increased, the growth of the tested yeasts decreased, and this decrease in multiplication was statistically significant for all tested strains at a glucose concentration of 30% compared to the initial analyzed glucose concentration of 1% *w*/*v* (Supplementary, Table S1, Figure S1).

It should be emphasized that the incubation conditions used were aerobic (shaking cultures); however, in *S. cerevisiae*, a yeast exhibiting a strong Crabtree effect, higher glucose concentrations induced fermentation processes [21]. In unconventional yeasts, due to their Crabtree-negative or petite-positive characteristics, glucose metabolism at higher sugar concentrations is directed towards multiplication processes, which was visible in the form of higher optical density values [22]. Moreover, *M. pulcherrima* yeasts known to be oleaginous microorganisms [23]. The ability to produce oil may contribute to a degree of osmotolerance in yeasts [24]. However, similar properties have been observed for the non-oleaginous yeast *W. anomalus*. Osmotolerance has often been described in this yeast [25,26,27]. It should therefore be assumed that the mechanisms of osmotolerance in yeasts may be very different. Cronwright et al. [28] studied the induction of glycerol production in yeast as a protective mechanism against an osmotic environment. It was noted that glycerol uptake and synthesis systems contribute to the osmotic tolerance of *Kluyveromyces marxianus* [29]. Dušková and co-workers similarly observed that glycerol uptake systems contribute to high osmotolerance in *Zygosaccharomyces rouxii* [30].

Numerous experiments have shown that yeast strains that are tolerant to high sugar concentrations effectively produce ethanol in media containing up to 25% *w*/*v* glucose [31]. Yeasts identified as *S. cerevisiae*, *M. pulcherrima*, and *W. anomalus* are often isolated from the nectar of flowering plants and sugar-rich food as spoiling microbiota [20,31,32,33]. *M. pulcherrima* is a good candidate for obtaining beverages with low ethanol content but interesting volatile profiles. *W. anomalus* strains can be used in oenology, due to their tolerance of up to 12.5% (*v*/*v*) ethanol [34]. However, *Brettanomyces*/*Dekkera bruxellensis* is a particularly troublesome wine spoilage yeast [35].

### 2.2. Fermentation Performance of Tested Yeast Strains

**Figure 1:**
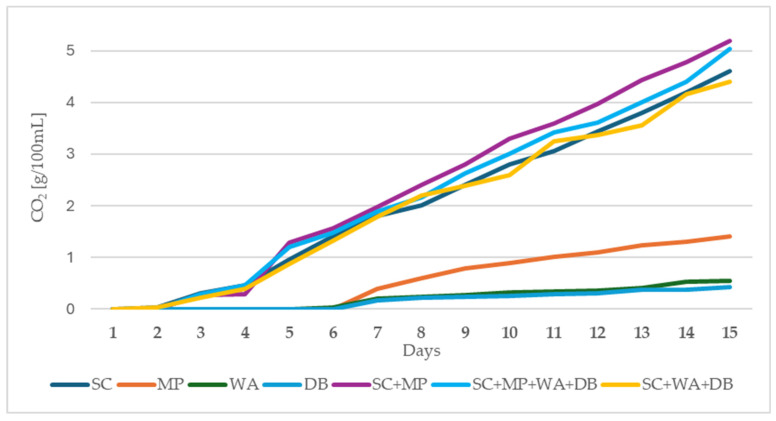
Fermentation performance (CO_2_ formation) of the tested yeast strains as monocultures and mixed populations. SC—*Saccharomyces cerevisiae*, MP—*Metschnikowia pulcherrima*, WA—*Wickerhamomyces anomalus*, DB—*Dekkera bruxellensis*.

Figure 1 shows CO_2_ production during fermentation trials. The highest fermentation rates were noted in the samples fermented with *S. cerevisiae*. As monocultures, the non-conventional strains showed poor fermentation dynamics during the first 15 days. *S. cerevisiae* started gas production on the second day after inoculation.

Unconventional strains started producing CO_2_ after the sixth day. The fermentation process was slow in all tested variants, even after 15 days of incubation. Interestingly, the population consisting of *S. cerevisiae* and *M. pulcherrima* cultures showed better fermentation performance than the *S. cerevisiae* monoculture. Similar dynamics characterized the mixed populations of SC + MP + DB + WA and SC + DB + WA. A statistical analysis of the obtained results is presented in Table S2 (Supplementary).

The use of non-*Saccharomyces* species as co-starter cultures with *S. cerevisiae* is becoming a common practice in the oenological industry. According to the literature, the growth of non-*Saccharomyces* yeasts can affect alcoholic fermentation by *S. cerevisiae* [36]. Such “cooperation” has been reported between *S. uvarum* and *M. pulcherrima* by Contreras et al. [9]. Mencher et al. [37] studied the transcriptional responses of *S. cerevisiae* to short-term co-cultivation with *M. pulcherrima* and other non-conventional yeasts. The results showed over-expression of the gluco-fermentative pathway, which was much stronger than with the other yeast species. Moreover, a strong repression of the respiration pathway was observed in response to *Metschnikowia* sp. The authors suggested that a direct interaction stress response may occur between *S. cerevisiae* and the other yeasts, which, under excess sugar conditions, induces transcription of the hexose transporters, triggers glucose flow towards fermentation, and inhibits respiration, leading to an increase in both metabolic flow and population dynamics. Recently, the results of transcriptomic analyses have confirmed interspecific communication between *S. cerevisiae* and *M. pulcherrima*. Mejias-Ortiz and co-workers [38] reported the upregulation of yeast metabolism in response to competing species. This finding points to the presence of signals that yeast cells may perceive as cues, indicating the presence of competitors.

### 2.3. HPLC Analysis

The honey worts fermented with mono- and co-cultures of yeasts were characterized by diverse chemical profiles (Table 2 and Table 3). The *S. cerevisiae* monoculture showed the best glucose and fructose consumption during the fermentation of honey broth. The monocultures of non-conventional yeasts, especially *W. anomalus* and *D. bruxelensis*, demonstrated the weakest sugar utilization.

The monoculture of *M. pulcherrima* was characterized by fairly good glucose consumption in the fermentation trials. Fructose was either barely utilized or not utilized by the non-conventional yeast monocultures. This indicates the common phenomenon of glucose repression, since fructose will not be metabolized when glucose is available [39]. A statistical analysis of the results is presented in Figure S3 (Supplementary).

The fermented honey wort after 15-day fermentation by a monoculture of *S. cerevisiae* contained 45.75 g/L ethanol and 5.73 g/L glycerol, with a residual sugar content of 175.36 g/L. The fermented alcoholic beverage obtained using an *M. pulcherrima* monoculture contained a significantly lower content of ethanol (12.781 g/L) and higher contents of glycerol (7.91 g/L) and residual sugars (248.97 g/L) in comparison to the beverage from *S. cerevisiae* (*p* > 0.05). The beverages obtained using other monocultures had the lowest ethanol content (10.53–12.49 g/L) but similar residual sugar content (318–319 g/L) and glycerol concentrations (1.4–1.9 g/L). Better results were obtained with mixed populations. These fermented honey broths contained higher levels of ethanol (57.7–63.9 g/L) and glycerol (7.03–7.16 g/L). The best results were noted for the SC + MP population (Supplementary, Table S3; Figure S4).

In all samples after fermentation, small amounts of methanol were detected. Yeasts are able to produce methanol through hydrolysis of pectins; alternatively, this compound may be synthesized from glycine [40,41,42]. In this case, the second option seems more likely because there is practically no pectin in honey, but glycine may be present in this source as one of several amino acids [42].

*M. pulcherrima* strains are widely used as starter strains in wine fermentation, both to improve the properties and for biocontrol in grape wines [43,44,45]. They can also be used as co-cultures for fruit wines. *M. pulcherrima* with *S. cerevisiae* produced wines with lower ethanol content and higher glycerol levels [13]. Glycerol is a major by-product of ethanol fermentation. Under anaerobic growth conditions, yeast cells produce glycerol to help maintain a cytosolic redox state conducive to sustaining glycolytic catabolism. In addition, glycerol has an important physiological function, because it is accumulated intracellularly when cells are exposed to decreased extracellular water activity [27,28]. Glycerol in wine can contribute to flavor intensity and aroma volatility [46,47]. Therefore, the increased production of glycerol has a positive effect both on the physiology of yeast cells and the sensory characteristics of alcoholic beverages. Our results obtained after HPLC analysis confirmed that honey broth is a suitable raw material for alcoholic fermentation by mixed cultures, especially those containing *M. pulcherrima* cells.

### 2.4. GC Analysis

*S. cerevisiae* and non-conventional yeasts presented different volatile characteristics. Their diverse secondary metabolic pathways and enzymatic profiles may contribute to the increased diversity of flavor phenotypes [13,47]. Our analysis of the main volatile compounds using GC-MS identified the main components of the volatilomes, with concentrations above 0.0001 mg/L (Supplementary, Table S3). The main compounds in the volatile profiles of all the obtained samples were as follows: acetaldehyde, propan-1-ol, ethyl acetate, 3-methylbutan-1-ol, and 2-methylbutan-1-ol. The lowest number of the major volatile compounds was obtained from the samples fermented with *D. bruxelensis* (8 compounds). The highest number was obtained for *M. pulcherrima* monoculture (13 compounds) and co-cultures SC + MP + DB + WA and SC + DB + WA (15–16 compounds). The samples fermented by *W. anomalus* and *S. cerevisiae* strains contained 9 and 11 compounds, respectively. However, despite having a greater variety of volatiles, the honey broth fermented with the co-cultures showed lower overall concentrations of volatiles (188.516–206.859 g/L) compared to the monoculture of *M. pulcherrima* (261.389 g/L).

The main volatile present in the highest concentrations was acetaldehyde. This metabolite was produced both by monocultures (3.535–138.881 g/L) and by co-cultures (35.761–47.128 g/L). The highest levels of acetaldehyde were recorded for the *M. pulcherrima* monoculture (138.881 g/L) and the co-culture with *S. cerevisiae* (SC + MP) (47.128 g/L). It is worth noting that the levels of this compound were significantly lower for honey broth fermented by mixed cultures. According to the literature, at low levels acetaldehyde can play a positive role in some specific aromatic contexts, while at higher levels it has negative effects associated with the generic presence of other aldehydes [48].

Another compound formed in higher amounts was ethyl acetate. This metabolite was detected especially in samples with *W. anomalus* and *M. pulcherrima* strains, both as monocultures (83.121 g/L and 32.536 g/L) and in co-cultures with the following strains: SC + MP + DB + WA (72.141 g/L) and SC + DB + WA (48.485 g/L). Ethyl acetate is an important component of the volatile profile, significantly contributing to the wine or mead aroma [48,49].

The following aliphatic higher alcohols were also found in the fermented honey samples: 3-methylbutan-1-ol, 2-methylpropan-1-ol, and 2-methylbutan-1-ol. The first compound, 3-methylbutan-1-ol, was produced by both the monoculture of *S. cerevisiae* (52.439 g/L) and co-cultures with this conventional yeast: SC + MP (56.083 g/L), SC + MP + DB + WA (61.214 g/L), and SC + DB + WA (65.180 g/L). 2-Methylpropan-1-ol was detected at the highest level in the samples fermented by the *M. pulcherrima* monoculture (25.038 g/L) as well as by the co-culture SC + MP + DB + WA (18.768 g/L). 2-Methylbutan-1-ol was produced mainly by the monocultures of *S. cerevisiae* (14.448 g/L) and *M. pulcherrima* (12.350 g/L), but it was also detected in the mixed fermentations of SC + MP (16.228 g/L), SC + MP + DB + WA (16.210 g/L), and SC + DB + WA (17.324 g/L). These aliphatic alcohols are often present in wines and meads and contribute desirable complexity to aroma at moderate concentrations. It is worth noting that concentrations of higher alcohols in the range of 300–400 mg/L are acceptable, but concentrations below 300 mg/L give a desirable, pleasant character [50,51]. Therefore, all the tested samples were within the limits of organoleptic acceptability. Unlike *S. cerevisiae* and other monocultures, the *M. pulcherrima* strain formed pentanal (0.027 g/L) but was not able to produce propanal, in contrast to DB (0.039 g/L) and WA (0.041 g/L). However, these aliphatic aldehydes were present in very small amounts in the fermented honey samples.

Principal component analysis (PCA) was performed to assess the effect of the yeast strains on the profile of volatile aromatic compounds in the analyzed samples. PCA allowed for the extraction of principal components (PCs) that significantly contributed to explaining the total variance of all the variables studied. A double criterion was used in the selection of PCs: the Kaiser criterion (eigenvalues of components > 1) and the criterion of the degree of explained variability >80%. The preliminary analysis showed that in the case of all fermentation variants using different yeast strains, the eigenvalues >1 were for the first three factors. Therefore, the components PC1, PC2, and PC3 were finally adopted as the three dimensions determining the space of the considered parameters (Table 4).

**Figure 2:**
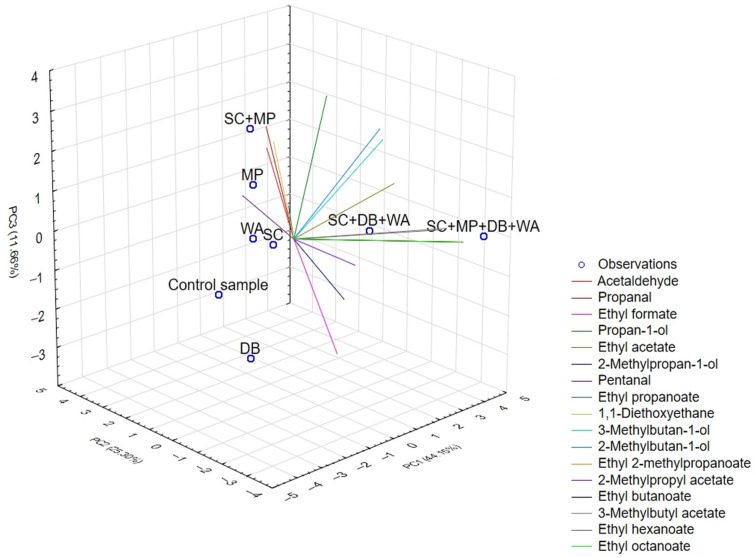
3D biplot of principal components PC1, PC2, and PC3; active observations—fermentation variants: SC, MP, DB, WA, SC + MP, SC + MP + DB + WA, SC + DB + WA, control sample. SC—*S. cerevisiae*, MP—*M. pulcherrima*, WA—*W. anomalus*, DB—*D. bruxellensis*.

The three principal components, PC1, PC2, and PC3, which are responsible in turn for the largest sources of variation in the data (81.1%), were used to prepare the plot space. Figure 2 shows a three-dimensional PCA biplot, incorporating both observations (samples) and variables (volatile compounds). The common visualization of variables and observations facilitates the inference of relationships between volatile compounds and the distribution of observations to each other, the identification of compounds that differentiate the tested samples to the greatest extent, and the identification of samples with a similar chemical profile.

The observations distributed in the three-dimensional plot demonstrated clear variation. The samples fermented by MP yeast and a mixed culture of all the strains employed in the study (SC + MP + DB + WA) exhibited a strong positive shift along PC1 and PC2, while the sample fermented by the three strains, i.e., SC + DB + WA, demonstrated a clear positive shift along PC1. These findings indicate that the significant influence of the variables highly correlated with these components and the unique aromatic profile of these samples concerning the other fermentation variants. Conversely, the sample fermented by the SC yeast strain exhibited a moderate shift toward positive PC1 and PC2 values, showing only partial similarity to the sample fermented by MP yeast. In contrast, the control sample, as well as the two samples fermented by the DB and WA yeast monocultures, are located in opposite areas of the plot, suggesting different volatile compound profiles.

The main component of PC1 strongly positively correlated with variables from the group of higher alcohols (propan-1-ol, 2-methylpropan-1-ol, 3-methylbutan-1-ol, and 2-methylbutan-1-ol) and esters (ethyl hexanoate, 3-methylbutyl acetate, ethyl 2-methylpropanoate, 2-methylpropyl acetate, ethyl hexanoate, ethyl octanoate, and ethyl butanoate). These are compounds with characteristic fruity notes. The samples fermented by MP monoculture and mixed cultures of SC + MP + DB + WA and SC + DB + WA yeasts correlated well with PC1, which indicates a rich profile of volatile compounds. The presence of ethyl esters and esters of higher alcohols should be considered particularly important, as they indicate the high technological or sensory potential of the yeast strains.

In turn, the PC2 showed a strong positive correlation with acetaldehyde and diethyl acetaldehyde acetal (1,1-diethoxyethane), explaining 92 and 86% of the variance of these two variables, respectively. The PC2 also positively correlated with ethyl propanoate, explaining 70% of the variance. An evaluation of the position of the observation representing the sample fermented by MP yeast revealed that, in comparison to other samples, it was distinguished by a higher concentration of these compounds.

A positive correlation was shown between PC3 and propanal, attributable to a four-times-higher concentration of this compound in samples fermented by a mixed culture of SC and MP yeasts compared to those fermented by DB, WA yeasts, and a mixed culture of SC + MP + DB + WA yeasts. On the other hand, in the case of ethyl acetate, the selected three components (PC1–PC3) explained a mere 41.6% of the variance in this variable, of which PC1 accounted for 22%. The correlation shown distinguishes, in terms of ethyl acetate concentration, fermentation variants that involve WA yeast, whether as a monoculture or in a mixed culture (SC + MP + DB + WA and SC + DB + WA).

In summary, PCA revealed differences between the yeast strains in terms of the profiles of volatile compounds. *M. pulcherrima* and mixed yeast cultures SC + DB + WA and SC + MP + DB + WA were characterized by high fermentation activity and the production of volatile compounds, including acetal (1,1-diethoxyethane), aldehydes (acetaldehyde and pentanal), higher alcohols (3-methylbutan-1-ol and 2-methylbutan-1-ol), and esters (ethyl propanoate, ethyl 2-methylpropanoate, 2-methylpropyl acetate, ethyl butanoate, 3-methylbutyl acetate, ethyl hexanoate, and ethyl octanoate).

Previous studies have shown that some non-conventional yeasts may also be used for mead production, with good results [50,52,53,54]. Non-*Saccharomyces* yeasts belonging to the *Torulaspora* genus used as pure cultures or mixed with *S. cerevisiae* increase the aromatic complexity of mead [53]. Mixed culture of different *Torulaspora* strains and *S. cerevisiae* show good fermentative performance in under 10 days. Recently, analysis of the chemical parameters of meads obtained by *S. cerevisiae* and other non-conventional strains of *Hansenula uvarum* in different combinations also showed differences between the samples in terms of residual sugars, acetic acid, glycerol, ethanol, and volatile organic compounds. Pleasant characteristics of sweetness, honeyness, and floralness were found in the mead fermented with co-cultures of *S. cerevisiae* and *H. uvarum*, while mead samples obtained using a monoculture of *S. cerevisiae* were dry, balanced, and free from foreign odors and tastes. These results show that the controlled use of conventional *S. cerevisiae* and non-conventional *H. uvarum* yeasts can be a promising approach to improving the quality of meads [54].

The potential of diverse non-*Saccharomyces* yeast strains applied in mead production is still unknown, which has greatly limited their use in practice [55,56]. Our research indicates that mixed fermentation using *S. cerevisiae* with *M. pulcherrima* and other non-conventional yeasts remarkably improves the sensory profiles of young meads. Metabolic analysis showed that *M. pulcherrima* had the greatest potential to improve the “balance” and “fullness” notes of young meads. These positive effects on the metabolic profiles of fermented young meads proved the important role of non-*S. cerevisiae* existing briefly in the early stages of fermentation.

## 3. Materials and Methods

### 3.1. Yeast Cultures

The conventional and non-conventional yeast strains used in this study are presented in Table 5. The yeasts strains were stored at −18 °C in a microbank storage system (Microbank^®^, Biomaxima, Lublin, Poland). A single bead was placed in wort broth (Merck Millipore, Darmstadt, Germany) to activate the strain. After 48 h of cultivation at 28 °C, one loop (10 μL) of yeast suspension was streaked onto potato dextrose agar (PDA) [potato extract 0.4% *w*/*v*; dextrose 2.0% *w*/*v*; agar 1.5% *w*/*v*] (Merck Millipore, Darmstadt, Germany) to ensure the purity of the yeast culture and incubated at 28 °C for two days.

### 3.2. Yeast Osmotolerance

To determine the growth profiles of the yeasts under varying glucose concentrations (0.5–30% *w*/*v*), YPD medium [yeast extract 2.5% *w*/*v*, peptone 5.0% *w*/*v*, glucose with variable concentrations] was used. The yeast inoculums were prepared in sterile Ringer solution. The optical density of the inoculum suspensions was 1.0 degrees according to the MacFarland scale (°McF), determined using a DEN-1 densitometer (Merck Millipore, Darmstadt, Germany). The intensity of yeast growth at 28 °C after 7 days in a rotary shaker (170 rpm) was measured using a DEN-1 densitometer and expressed on the McFarland scale.

### 3.3. Honey Wort Preparation

Multiflorous honey was obtained from a local beekeeper from the Malopolska region (Poland) and delivered by Datan Sp. z o.o. (Krakow, Poland) (50°03′41″ N, 19°56′18″ E). The honey was diluted with sterile distilled water in a volumetric proportion of 1:2 (honey:water). Sugar content was measured refractometrically (Anton Paar 6000 densimeter, Graz, Austria) and standardized to 36 °Bx. The mead wort was topped up with the addition of (NH_4_)_2_HPO_4_ (0.04% *w*/*v*), which is a popular supplement in mead-making [56]. The honey wort was gently heated (90 °C) for 30 min. The foam formed during heat treatment was systematically removed. After cooling, the specific gravity was controlled and set to 35 °Bx with sterile distilled water.

### 3.4. Fermentation Trials

The following yeast strains as monocultures and co-cultures were used in the experiments (Table 6).

Sterile Erlenmeyer flasks (volume 100 mL) were filled with 50 mL of the pasteurized honey wort. All samples were inoculated with 2.5 mL of standardized (6 °McF) yeast suspension (5% *v*/*v*). Mixed populations were prepared in volumetric proportions of 1:1 (two strains), 1:1:1 (three strains), or 1:1:1:1 (four strains). The flasks were closed with fermentation airlocks and silicone stoppers to allow CO_2_ to escape. The fermentation samples were incubated without agitation at 25 °C. The weight loss of the flasks due to the release of CO_2_ was monitored every day during the 2-week fermentation period. This fermentation time was chosen because non-*Saccharomyces* yeasts play a substantial role in the early stages of wine fermentation [57,58,59]. After fermentation, the samples were centrifuged (10 °C, 10,000× *g*, 10 min) using a centrifuge 5804R (Eppendorf, Wesseling-Berzdorf, Germany). The supernatant was collected and analyzed using chromatographic techniques. Prior to chromatography, clear liquid samples were prepared by filtration using 0.45 μm polyethersulfone membranes (Merck Millipore, Darmstadt, Germany).

### 3.5. HPLC Analysis

The profiles of the main saccharides, acetic acid, glycerol, methanol, and ethanol in the young meads were determined using an HPLC (Agilent 1260 Infinity, Agilent Technologies, Santa Clara, CA, USA) with a Hi-Plex H column (7.7 × 300 mm, 8 m, Agilent Technologies, Santa Clara, CA, USA) and a refractive index detector at 55 °C. The column temperature was maintained at 60 °C. As a mobile phase, a 5 mM solution of H_2_SO_4_ was used at a flow rate of 0.7 mL/min with a sample volume of 20 L [13,60]. The samples were analyzed as received and after 10 rounds of dilution with ultrapure water. The data were processed using OpenLab CDS Chemstation software Rev. C.01.06 (Agilent Technologies, Santa Clara, CA, USA).

Standard solutions of pure reagents in ultrapure water were prepared to quantify the concentration of the analyzed compounds in the range of 1.5–30.0 g/L for glucose, fructose, and ethanol; 0.04–9.2 g/L for glycerol; and 0.02–1.25 g/L for acetic acid. The linearity of the obtained calibration curves was satisfactory across the whole tested range, with an R2 value of at least 0.9997. The limit of detection (LOD) and limit of quantification (LOQ) were calculated according to the method proposed by Haubax and Vos [61].

### 3.6. GC-MS Analysis

To identify and quantify the volatiles in the obtained fermented honey samples, we used an Agilent 7890A GC (Agilent Technologies, Santa Clara, CA, USA) gas chromatograph equipped with an Agilent MSD 5975C quadrupole mass spectrometer and an Agilent 7697A headspace analyzer. The headspace sampler was connected to the gas chromatograph via a transfer line through the split–splitless injector. An Rxi-5 ms capillary column (60 m, 0.25 mm, 0.25 m; Restek, Bellefonte, PA, USA) was used to separate the compounds. The initial GC oven temperature was set to 30 °C and held for 6 min, then ramped up by 5 °C/min to 80 °C (held for 3 min), and ramped up again 10 °C/min to 230 °C. This final temperature was maintained for 6 min. The carrier gas was helium, with a flow rate of 1.2 mL/min. Before analysis, a 20 mL headspace vial was filled with a 7 mL sample of wine and closed tightly. The headspace conditions were as follows: the temperatures of the oven, loop, and transfer line were set at 50 °C, 60 °C, and 70 °C, respectively. The equilibration time and injection duration were 20 min and 0.7 min, respectively. During sample equilibration, the vial was shaken (136 shakes/min). The temperature of the injector was 250 °C. Injections were made in split mode (10:1). The temperatures of the MSD ion source, transfer line, and quadrupole were 230 °C, 250 °C, and 150 °C, respectively. The ionization energy was 70 eV.

Qualitative analysis was initially performed in full scan ion monitoring mode (SCAN). The volatile compounds in the young mead samples were identified by comparing their mass spectra with those of standard compounds and with the mass spectra of the NIST/EPA/NIH Mass Spectra Library (Version 2.0g). Next, quantitative analysis of the volatile compounds in the tested samples was performed using the external calibration method. Quantitative analysis was performed in selected ion monitoring mode (SIM). The calibration standards were prepared using a dilution series of an external analytical standard mixture. The linearity of the calibration curves was tested over the following concentration ranges: 2–100 mg/L for ethyl acetate and acetaldehyde, 1–200 mg/L for 3-methylbutan-1-ol and 2-methylbutan-1-ol, 0.5–100 mg/L for 2-methylpropan-1-ol, 0.25–10 mg/L for propan-1-ol, 0.005–0.25 mg/L for propanal and pentanal, 0.1–1 mg/L for furan-2-carbaldehyde, 0.1–5 mg/L for butane-2,3-dione and 3-methylfuran, 0.005–1 mg/L for 1,1-diethoxyethane, 0.01–5 mg/L for ethyl formate, 0.01–25 mg/L for methyl acetate, 2–50 g/L for pentan-2one, and 0.5–100 g/L for other esters (ethyl propanoate, ethyl-2-methylpropanoate, 2-methylpropyl acetate, ethyl butanoate, 2-methylbutyl acetate, 3-methylbutyl acetate, ethyl hexanoate, ethyl octanoate, ethyl decanoate, ethyl 2-methylbutanoate, and ethyl-3-methylbutanoate). The correlation coefficients of the calibration curves of the external standards were 0.99 on average. The values of LOD and LOQ were calculated based on the standard deviation of the response and the slope of the calibration curve at levels approximating the LOD [61]. The obtained data were analyzed using Agilent MassHunter software B.07.00 (Agilent Technologies, Santa Clara, CA, USA).

### 3.7. Statistics

The results of the statistical analysis were presented as the mean ± SD of three separate experiments (each variant of wine was prepared in three replicates, with one technical sample). As the results did not follow a normal distribution (Shapiro–Wilk test), non-parametric tests were used for the statistical analysis of the following experiments: yeast strain growth profiles under varying glucose concentrations, weight loss during the fermentation process, and changes in the chemical composition of the wort. Differences in the analyzed parameters were determined using the Kruskal–Wallis test (KW test), followed by a multiple comparisons test (MCT) to indicate significant differences between the groups. A *p*-value < 0.05 was considered statistically significant. The KW, MCT, and independent sample *t*-Tests were performed using Statistica^®^13.1 (StatSoft, Tulsa, OK, USA). In addition, to investigate the ability of different yeast strains used as monocultures and mixed cultures to synthesize volatile compounds during fermentation, principal component analysis (PCA) was performed with Statistica^®^13.3 (TIBCO Software Inc., San Ramon, CA, USA).

## 4. Conclusions

In this study, we investigated the effects of using conventional *S. cerevisiae* and non-conventional yeasts as starter cultures in controlled mixed fermentations of honey wort. Various species of unconventional yeast were selected based on their physiology and fermentation characteristics. Particular attention was paid to the yeast *M. pulcherrima*, due to the growing interest in this yeast in winemaking. The obtained profiles showed that mixed cultures strongly altered the aromatic profiles of fermented honey samples in comparison to the corresponding monocultures. In general, the aromatic complexity of the fermented honey wort was improved by using non-conventional yeasts, especially the *M. pulcherrima* strain, as a co-starter. After co-inoculation with *S. cerevisiae*, the young mead showed a lower ethanol content, higher glycerol level, and higher concentration of volatile substances. Inoculation with other unconventional yeasts from the genera *Dekkera* and *Wickerhamomyces* did not change this beneficial effect. This preliminary study is a first step towards the preparation of honey worts using conventional and non-conventional yeasts, and evaluation of their chemical properties after fermentation. Future studies will investigate different yeast strains, inoculation methods, and honey broth supplements, which may require longer fermentation times. We also plan to explore a wider range of honey varieties and conduct larger-scale fermentations.

## Figures and Tables

**Table 1:** Yeast growth [°McF] with different concentrations of glucose [% *w*/*v*] in the culture medium. The results are presented as the mean ± SD. Statistically significant differences in growth (KW test, followed by MCT) are indicated in bold, The *p*-value is given below the data for each strain. The letters indicate between which strains there is a statistically significant difference.

Yeast Strain	Glucose Concentration in the Culture Medium [% *w*/*v*]			
	1	10	20	30
*S. cerevisiae*	8.967 ± 0.252	7.600 ± 0.458	4.867 ± 0.306	3.567 ± 0.321
*M. pulcherrima*	8.367 ± 0.208	7.933 ± 0.551	**6.667 ± 0.321 ^A^**	**5.900 ± 0.100 ^B^**
*D. bruxellensis*	8.067 ± 0.493	6.767 ± 0.321	**3.833 ± 0.153 ^A^**	**2.867 ± 0.153 ^B^**
*W. anomalus*	8.967 ± 0.115	7.867 ± 0.208	6.233 ± 0.416	4.167 ± 0.404
*p* value	*p* > 0.05	*p* > 0.05	**^A^** 0.019	**^B^** 0.013

**Table 2:** Glucose and fructose content in honey worts fermented by monocultures and mixed populations. The control sample is the honey wort before fermentation. The results are presented as the mean ± SD. Statistically significant differences (KW test, followed by MCT) are indicated in bold. The letters indicate between which groups there is a statistically significant difference.

Yeasts	Sample	Glucose [g/L]	Fructose [g/L]
	Control	182.466 ± 6.513	189.375 ± 8.407
Monocultures	SC *	**65.555 ± 1.092 ^A^**	**110.476 ± 5.472 ^B^**
	MP	89.211 ± 2.321	159.559 ± 10.990
	WA	145.564 ± 4.127	**173.857 ± 5.550 ^B^**
	DB	**152.324 ± 4.052 ^A^**	166.734 ± 4.202
Mixed populations	SC + MP	70.123 ± 3.830	128.480 ± 2.703
	SC + MP + WA + DB	79.390 ± 2.683	130.322 ± 6.252
	SC + WA + DB	73.015 ± 2.804	126.511 ± 2.984
*p*-value		*p* = 0.013	*p* = 0.013

* SC—*S. cerevisiae*, MP—*M. pulcherrima*, WA—*W. anomalus*, DB—*D. bruxellensis*.

**Table 3:** The metabolic profiles in fermented honey wort obtained by monocultures and co-cultures of yeasts. The results are presented as the mean ± SD. The control sample is the honey wort before fermentation. Statistically significant differences (KW test, followed by MCT) in compound content are indicated in bold. The letters indicate between which groups there is a statistically significant difference.

Yeast Strains		Compound [g/L]			
		Glycerol	Acetic Acid	Methanol	Ethanol
Control		0.197 ± 0.006	0.000 ± 0.000	0.000 ± 0.000	0.000 ± 0.000
Monocultures	SC *	5.762 ± 0.345	0.858 ± 0.016	**2.281 ± 0.103 ^C^**	45.563 ± 0.903
	MP	**7.928 ± 0.178 ^A^**	0.562 ± 0.011	1.247 ± 0.193	12.781 ± 0.547
	WA	1.873 ± 0.054	0.387 ± 0.007	0.153 ± 0.017	12.442 ± 0.586
	DB	**1.362 ± 0.057 ^A^**	**0.227 ± 0.010 ^B^**	**0.126 ± 0.007 ^C^**	**10.495 ± 0.497 ^D^**
Mixed populations	SC + MP	7.155 ± 0.046	0.928 ± 0.026	1.242 ± 0.122	**63.511 ± 1.948 ^D^**
	SC + MP + WA + DB	7.107 ± 0.097	**1.087 ± 0.028 ^B^**	2.081 ± 0.085	57.630 ± 1.377
	SC + WA + DB	7.019 ± 0.186	0.944 ± 0.043	1.241 ± 0.119	59.549 ± 1.899
*p*-value		0.008	0.008	0.008	0.008

* SC—*S. cerevisiae*, MP—*M. pulcherrima*, WA—*W. anomalus*, DB—*D. bruxellensis*.

**Table 4:** Eigenvalues with % of the total variance explained by the principal components.

Principal Component	Eigenvalue	Variability [%]	Cumulative [%]
PC1	7.505	44.147	44.147
PC2	4.301	25.298	69.445
PC3	1.982	11.658	81.103

**Table 5:** Yeast strains used in the study.

Strain	Origin	Strain Abbreviation	References
*Saccharomyces cerevisiae* Tokay LOCK0203	LOCK *	SC	[13,20]
*Metschnikowia pulcherrima* NCYC747	NCYC **	MP	[13,20]
*Dekkera bruxellensis* NCYC D5300	NCYC	DB	[13,20]
*Wickerhamomyces anomalus* NCYC D5299	NCYC	WA	[13,20]

* Collection of Pure Cultures of Industrial Microorganisms, Lodz University of Technology, Poland, ** National Collection of Yeast Cultures; Norwich, United Kingdom.

**Table 6:** Yeast cultures used for fermentation trials.

Monocultures	Mixed Populations
*Saccharomyces cerevisiae* SC	*S. cerevisiae* SC + *M. pulcherrima* MP
*Metschnikowia pulcherrima* MP	*S. cerevisiae* SC + *M. pulcherrima* MP + *W. anomalus* WA + *D. bruxellensis* DB
*Dekkera bruxellensis* DB	
*Wickerhamomyces anomalus* WA	*S. cerevisiae* SC + *W. anomalus* WA + *D. bruxellensis* DB

## Data Availability

The original contributions presented in this study are included in the article. Further inquiries can be directed to the corresponding author.

## Supplementary Materials

The following supporting information can be downloaded at: https://www.mdpi.com/article/10.3390/molecules30112319/s1, Figure S1: Box-and-whisker plots showing mean yeast growth values at 20% and 30% glucose concentrations; Figure S2: Box-and-whisker plots showing mean CO_2_ formation after 7- and 15-day fermentation; Figure S3: Box-and-whisker plots showing glucose and fructose content in monocultures and mixed populations; Figure S4: Box-and-whisker plots showing the compound content (glycerol, acetic acid, methanol, ethanol) in fermented honey beverages produced by tested yeast strains as monocultures and mixed populations; Table S1: The comparison of yeast growth depending on the glucose concentration in the culture medium; Table S2: Fermentation performance of tested yeast strains as monocultures and mixed populations; Table S3: Volatilomes of honey wort fermented by monocultures and mixed populations.

## References

[1] Bisson L.F. (1999). Stuck and sluggish fermentations. AJEV.

[2] Binati R.L., Lemos Junior W.J.F., Luzzini G., Slaghenaufi D., Ugliano M., Torriani S. (2020). Contribution of non-*Saccharomyces* yeasts to wine volatile and sensory diversity: A study on *Lachancea thermotolerans*, *Metschnikowia* spp. and *Starmerella bacillaris* strains isolated in Italy. Int. J. Food Microbiol..

[3] Vicente J., Ruiz J., Belda I., Benito-Vázquez I., Marquina D., Calderón F., Santos A., Benito S. (2020). The genus *Metschnikowia* in enology. Microorganisms.

[4] Zhang B., Tang C., Yang D., Liu H., Xue J., Duan C., Yan G. (2022). Effects of three indigenous non-*Saccharomyces* yeasts and their pairwise combinations in co-fermentation with *Saccharomyces cerevisiae* on volatile compounds of Petit Manseng wines. Food Chem..

[5] Sipiczki M. (2016). Overwintering of vineyard yeasts: Survival of interacting yeast communities in grapes mummified on vines. Front. Microbiol..

[6] Morata A., Escott C., Bañuelos M.A., Loira I., Fresno J.M.D., González C., Suárez-Lepe J.A. (2019). Contribution of non-*Saccharomyces* yeasts to wine freshness. A review. Biomolecules.

[7] Sipiczki M. (2020). *Metschnikowia pulcherrima* and related pulcherrimin-producing yeasts: Fuzzy species boundaries and complex antimicrobial antagonism. Microorganisms.

[8] Tufariello M., Fragasso M., Pico J., Panighel A., Castellarin S.D., Flamini R., Grieco F. (2021). Influence of non-*Saccharomyces* on wine chemistry: A focus on aroma-related compounds. Molecules.

[9] Contreras A., Curtin C., Varela C. (2015). Yeast population dynamics reveal a potential ‘collaboration’ between *Metschnikowia pulcherrima* and *Saccharomyces uvarum* for the production of reduced alcohol wines during Shiraz fermentation. Appl. Microbiol. Biotechnol..

[10] Morales P., Rojas V., Quirós M., Gonzalez R. (2015). The impact of oxygen on the final alcohol content of wine fermented by a mixed starter culture. Appl. Microbiol. Biotechnol..

[11] Mencher A., Morales P., Valero E., Tronchoni J., Patil K.R., Gonzalez R. (2020). Proteomic characterization of extracellular vesicles produced by several wine yeast species. Microb. Biotechnol..

[12] Castrillo D., Blanco P. (2023). Characterization of indigenous non-*Saccharomyces* yeast strains with potential use in winemaking. Front. Biosci..

[13] Kregiel D., Pawlikowska E., Antolak H., Dziekonska-Kubczak U., Pielech-Przybylska K. (2022). Exploring use of the *Metschnikowia pulcherrima* clade to improve properties of fruit wines. Fermentation.

[14] Puyo M., Simonin S., Bach B., Klein G., Alexandre H., Tourdot-Maréchal R. (2023). Bio-protection in oenology by *Metschnikowia pulcherrima*: From field results to scientific inquiry. Front. Microbiol..

[15] Altieri V., Rossi V., Fedele G. (2023). Efficacy of preharvest application of biocontrol agents against gray mold in grapevine. Front. Plant Sci..

[16] Ramalhosa E., Gomes T., Pereira A.P., Dias T., Estevinho L.M. (2011). Mead production: Tradition versus modernity. Adv. Food Nutr. Res..

[17] Iglesias A., Pascoal A., Choupina A.B., Carvalho C.A., Feás X., Estevinho L.M. (2014). Developments in the fermentation process and quality improvement strategies for mead production. Molecules.

[18] Webster C.E., Barker D., Deed R.C., Pilkington L.I. (2025). Mead production and quality: A review of chemical and sensory mead quality evaluation with a focus on analytical methods. Food Res. Int..

[19] Pereira A.P., Dias T., Andrade J., Ramalhosa E., Estevinho L.M. (2009). Mead production: Selection and characterization assays of *Saccharomyces cerevisiae* strains. Food Chem. Toxicol..

[20] Pawlikowska E., James S.A., Breierova E., Antolak H., Kregiel D. (2019). Biocontrol capability of local *Metschnikowia* sp. isolates. Antonie Van Leeuwenhoek.

[21] Imura M., Nitta K., Iwakiri R., Matsuda F., Shimizu H., Fukusaki E. (2020). Comparison of metabolic profiles of yeasts based on the difference of the Crabtree positive and negative. J. Biosci. Bioeng..

[22] Blomqvist J., Passoth V. (2015). *Dekkera bruxellensis*-spoilage yeast with biotechnological potential, and a model for yeast evolution, physiology and competitiveness. FEMS Yeast Res..

[23] Němcová A., Szotkowski M., Samek O., Cagáňová L., Sipiczki M., Márová I. (2021). Use of waste substrates for the lipid production by yeasts of the genus *Metschnikowia*-screening study. Microorganisms.

[24] Breuer U., Harms H. (2006). *Debaryomyces hansenii*-an extremophilic yeast with biotechnological potential. Yeast.

[25] Niu C., Yuan Y., Hu Z., Wang Z., Liu B., Wang H., Yue T. (2016). Accessing spoilage features of osmotolerant yeasts identified from kiwifruit plantation and processing environment in Shaanxi, China. Int. J. Food Microbiol..

[26] Thammaket J., Srimongkol P., Ekkaphan P., Thitiprasert S., Niyomsin S., Chaisuwan T., Chirachanchai S., Thongchul N. (2024). Isolation, screening, and characterization of the newly isolated osmotolerant yeast *Wickerhamomyces anomalus* BKK11-4 for the coproduction of glycerol and arabitol. Braz. J. Microbiol..

[27] Stratford M., Steels H., Novodvorska M., Archer D.B., Avery S.V. (2019). Extreme osmotolerance and halotolerance in food-relevant yeasts and the role of glycerol-dependent cell individuality. Front. Microbiol..

[28] Cronwright G.R., Rohwer J.M., Prior B.A. (2002). Metabolic control analysis of glycerol synthesis in *Saccharomyces cerevisiae*. Appl. Environ. Microbiol..

[29] Zhang B., Ren L., Wang H., Xu D., Zeng X., Li F. (2020). Glycerol uptake and synthesis systems contribute to the osmotic tolerance of *Kluyveromyces marxianus*. Enzyme Microb. Technol..

[30] Dušková M., Ferreira C., Lucas C., Sychrová H. (2015). Two glycerol uptake systems contribute to the high osmotolerance of *Zygosaccharomyces rouxii*. Mol. Microbiol..

[31] Chen A., Qu T., Smith J.R., Li J., Du G., Chen J. (2024). Osmotic tolerance in *Saccharomyces cerevisiae*: Implications for food and bioethanol industries. Food Biosci..

[32] Siavoshi F., Sahraee M., Heydari S., Sarrafnejad A., Saniee P., Tavakolian A., Heidarian S. (2020). Sugar-rich foods carry osmotolerant yeasts with intracellular *Helicobacter pylori* and *Staphylococcus* spp. Middle East J. Dig. Dis..

[33] Tiwari S., Jadhav R., Avchar R., Lanjekar V., Datar M., Baghela A. (2021). Nectar yeast community of tropical flowering plants and assessment of their osmotolerance and xylitol-producing potential. Curr. Microbiol..

[34] Maicas S., Mateo J.J. (2023). The life of *Saccharomyces* and non-*Saccharomyces* yeasts in drinking wine. Microorganisms.

[35] Romano A., Perello M.C., de Revel G., Lonvaud-Funel A. (2008). Growth and volatile compound production by *Brettanomyces*/*Dekkera bruxellensis* in red wine. J. Appl. Microbiol..

[36] Aplin J.J., White K.P., Edwards C.G. (2019). Growth and metabolism of non-*Saccharomyces* yeasts isolated from Washington state vineyards in media and high sugar grape musts. Food Microbiol..

[37] Mencher A., Morales P., Curiel J.A., Gonzalez R., Tronchoni J. (2021). *Metschnikowia pulcherrima* represses aerobic respiration in *Saccharomyces cerevisiae* suggesting a direct response to co-cultivation. Food Microbiol..

[38] Mejias-Ortiz M., Mencher A., Morales P., Tronchoni J., Gonzalez R. (2023). *Saccharomyces cerevisiae* responds similarly to co-culture or to a fraction enriched in *Metschnikowia pulcherrima* extracellular vesicles. Microb. Biotechnol..

[39] Carlson M. (1999). Glucose repression in yeast. Curr. Opin. Microbiol..

[40] Blumenthal P., Steger M.C., Einfalt D., Rieke-Zapp J., Quintanilla Bellucci A., Sommerfeld K., Schwarz S., Lachenmeier D.W. (2021). Methanol mitigation during manufacturing of fruit spirits with special consideration of novel coffee cherry spirits. Molecules.

[41] Shen J., Huang W., You Y., Zhan J. (2024). Controlling strategies of methanol generation in fermented fruit wine: Pathways, advances, and applications. Compr. Rev. Food Sci. Food Saf..

[42] Yang J., Liu Y., Cui Z., Wang T., Liu T., Liu G. (2024). Analysis of free amino acid composition and honey plant species in seven honey species in China. Foods.

[43] Oro L., Ciani M., Comitini F. (2014). Antimicrobial activity of *Metschnikowia pulcherrima* on wine yeasts. J. Appl. Microbiol..

[44] Canonico L., Comitini F., Ciani M. (2019). *Metschnikowia pulcherrima* selected strain for ethanol reduction in wine: Influence of cell immobilization and aeration condition. Foods.

[45] Binati R.L., Maule M., Luzzini G., Martelli F., Felis G.E., Ugliano M., Torriani S. (2023). From bioprotective effects to diversification of wine aroma: Expanding the knowledge on *Metschnikowia pulcherrima* oenological potential. Food Res. Int..

[46] Tapia S.M., Cuevas M., Abarca V., Delgado V., Rojas V., García V., Brice C., Martínez C., Salinas F., Larrondo L.F. (2018). GPD1 and ADH3 natural variants underlie glycerol yield differences in wine fermentation. Front. Microbiol..

[47] Carrau F., Boido E., Dellacassa E., Merillon J.M., Ramawat K.G. (2017). Yeast diversity and flavor compounds. Fungal Metabolities.

[48] Arias-Pérez I., Sáenz-Navajas M.P., De-La-Fuente-Blanco A., Ferreira V., Escudero A. (2021). Insights on the role of acetaldehyde and other aldehydes in the odour and tactile nasal perception of red wine. Food Chem..

[49] Yu H., Xie T., Xie J., Ai L., Tian H. (2019). Characterization of key aroma compounds in Chinese rice wine using gas chromatography-mass spectrometry and gas chromatography-olfactometry. Food Chem..

[50] Felipe A.L.D., Souza C.O., Santos L.F., Cestari A. (2019). Synthesis and characterization of mead: From the past to the future and development of a new fermentative route. J. Food Sci. Technol..

[51] De-La-Fuente-Blanco A., Saenz-Navajas M.P., Ferreira V. (2016). On the effects of higher alcohols on red wine aroma. Food Chem..

[52] Qureshi N., Tamhane D.V. (1987). Production of mead by immobilized cells of *Hansenula anomala*. Appl. Microbiol. Biotechnol..

[53] Barry J.P., Metz M.S., Hughey J., Quirk A., Bochman M.L. (2018). Two novel strains of *Torulaspora delbrueckii* isolated from the honey bee microbiome and their use in honey fermentation. Fermentation.

[54] Prestianni R., Matraxia M., Naselli V., Pirrone A., Badalamenti N., Ingrassia M., Gaglio R., Settanni L., Columba P., Maggio A. (2022). Use of sequentially inoculation of *Saccharomyces cerevisiae* and *Hanseniaspora uvarum* strains isolated from honey by-products to improve and stabilize the quality of mead produced in Sicily. Food Microbiol..

[55] Liu Y., Jiang B., Wang K. (2023). A review of fermented bee products: Sources, nutritional values, and health benefits. Food Res. Int..

[56] Czabaj S., Kawa-Rygielska J., Kucharska A.Z., Kliks J. (2017). Effects of mead wort heat treatment on the mead fermentation process and antioxidant activity. Molecules.

[57] García M., Greetham D., Wimalasena T.T., Phister T.G., Cabellos J.M., Arroyo T. (2016). The phenotypic characterization of yeast strains to stresses inherent to wine fermentation in warm climates. J. Appl. Microbiol..

[58] Holt S., Mukherjee V., Lievens B., Verstrepen K.J., Thevelein J.M. (2018). Bioflavoring by non-conventional yeasts in sequential beer fermentations. Food Microbiol..

[59] Cioch-Skoneczny M., Satora P., Skoneczny S., Skotniczny M. (2021). Biodiversity of yeasts isolated during spontaneous fermentation of cool climate grape musts. Arch. Microbiol..

[60] Dziekońska-Kubczak U., Berłowska J., Dziugan P., Patelski P., Pielech-Przybylska K., Balcerek M. (2018). Nitric acid pretreatment of Jerusalem artichoke stalks for enzymatic saccharification and bioethanol production. Energies.

[61] Hubaux A., Vos G. (1970). Decision and detection limits for calibration curves. Anal. Chem..

