# Supplementary material for: Chemical Fingerprints of Honey Fermented by Conventional and Non-Conventional Yeasts
Source: Molecules. 2025 May 26;30(11):2319. doi: 10.3390/molecules30112319 (PMC12156296; doi:10.3390/molecules30112319)
Supplement: Supplementary file 1 [file molecules-30-02319-s001.zip › molecules-3649418-supplementary.pdf]

Chemical fingerprints of honey fermented by conventional and non-conventional yeasts

Dorota Kregiel, Urszula Dziekonska-Kubczak, Karolina Czarnecka- Chrebelska, and Katarzyna Pielech-Przybylska

Supplementary

Table S1. The comparison of yeast growth [°McF] depending on the glucose concentration in the culture medium. Statistically significant differences (KW test, followed by MCT) in growth are indicated in bold with *. The p-value is given on the right for each strain.

Yeast strain	Glucose concentration [%]				p value (KW test, followed by MCT)
	1	10	20	30	
S. cerevisiae	**8.967 ± 0.252***	7.600 ± 0.458	4.867 ± 0.306	**3.567 ± 0.321***	0.013
M. pulcherrima	**8.367 ± 0.208***	7.933 ± 0.551	6.667 ± 0.321	**5.900 ± 0.100***	0.028
D. bruxellensis	**8.067 ± 0.493***	6.767 ± 0.321	3.833 ± 0.153	**2.867 ± 0.153***	0.013
W. anomalus	**8.967 ± 0.115***	7.867 ± 0.208	6.233 ± 0.416	**4.167 ± 0.404***	0.013

With the increasing glucose concentrations *M. pulcherrima* showed higher osmotolerance, with a notable increase in 30% glucose concentration in comparison to other tested strains. The increase was statistically significant compared to *D. bruxellensis* in 20% and 30% glucose concentrations (p=0.019; p=0.013, respectively, K-W test).

Figure S1. Box and whisker plots showing mean yeast growth [°McF] values at 20% and 30% glucose concentrations. The growth of *M. pulcherrima* was statistically significant compared to *D. bruxellensis* growth in 20% and 30% glucose levels ($p=0.019$; $p=0.013$, respectively, K-W test).

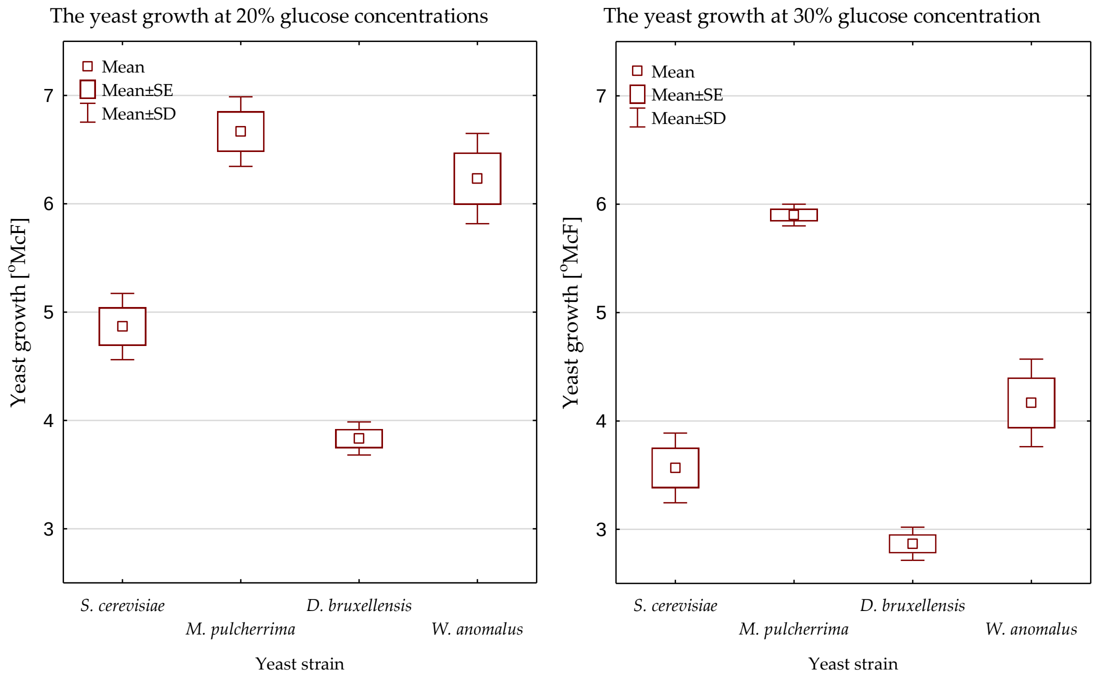

Table S2. Fermentation performance (CO_2 formation) of tested yeast strains as monocultures and mixed populations. The results are presented as the mean ± SD. Statistically significant differences (KW test, followed by MCT) in CO_2 production are indicated in bold.

Day of fermentation	Monocultures				Mixed populations			P value
	**SC**	**MP**	**WA**	**DB**	**SC+MP**	**SC+MP+ WA+DB**	**SC+WA+ DB**	
1	0.000 ± 0.000	0.000 ± 0.000	0.000 ± 0.000	0.000 ± 0.000	0.000 ± 0.000	0.000 ± 0.000	0.000 ± 0.000	
2	0.033 ± 0.058	0.000 ± 0.000	0.000 ± 0.000	0.000 ± 0.000	0.000 ± 0.000	0.000 ± 0.000	0.033 ± 0.058	
3	0.300 ± 0.010	0.000 ± 0.000	0.000 ± 0.000	0.000 ± 0.000	0.263 ± 0.006	0.283 ± 0.015	0.220 ± 0.010	
4	0.457 ± 0.021	0.000 ± 0.000	0.000 ± 0.000	0.000 ± 0.000	0.280 ± 0.010	0.460 ± 0.010	0.390 ± 0.010	
5	0.953 ± 0.047	0.000 ± 0.000	0.000 ± 0.000	0.000 ± 0.000	1.277 ± 0.025	1.200 ± 0.000	0.873 ± 0.025	
6	1.400 ± 0.020	0.000 ± 0.000	0.033 ± 0.058	0.000 ± 0.000	1.563 ± 0.015	1.475 ± 0.015	1.327 ± 0.006	
7	1.800 ± 0.100	0.383 ± 0.076	0.203 ± 0.025	**0.163 ± 0.055[A]**	**1.967 ± 0.153[A]**	1.883 ± 0.015	1.780 ± 0.020	**[A] p=0.047**
8	2.017 ± 0.076	0.603 ± 0.015	**0.240 ± 0.010[A]**	**0.217 ± 0.032[B]**	**2.400 ± 0.100[A,B]**	2.167 ± 0.058	2.200 ± 0.100	**[A]p=0.047; [B]p=0.015**
9	2.400 ± 0.020	0.793 ± 0.015	0.277 ± 0.012	**0.240 ± 0.030[A]**	**2.797 ± 0.006[A]**	2.633 ± 0.058	2.393 ± 0.090	**[A]p=0.010**
10	2.800 ± 0.026	0.897 ± 0.015	0.323 ± 0.015	**0.257 ± 0.021[A]**	**3.297 ± 0.100[A]**	3.000 ± 0.000	2.600 ± 0.100	**[A]p=0.008**
11	3.057 ± 0.125	1.017 ± 0.076	0.340 ± 0.010	**0.280 ± 0.010[A]**	**3.600 ± 0.100[A]**	3.417 ± 0.076	3.243 ± 0.051	**[A]p=0.009**
12	3.433 ± 0.115	1.093 ± 0.110	0.360 ± 0.010	**0.300 ± 0.010[A]**	**3.967 ± 0.153[A]**	3.610 ± 0.010	3.367 ± 0.058	**[A]p=0.008**
13	3.800 ± 0.100	1.233 ± 0.058	0.400 ± 0.010	**0.363 ± 0.015[A]**	**4.443 ± 0.140[A]**	4.000 ± 0.000	3.560 ± 0.164	**[A]p=0.008**
14	4.193 ± 0.042	1.300 ± 0.010	0.523 ± 0.023	**0.377 ± 0.015[A]**	**4.783 ± 0.076[A]**	4.400 ± 0.100	4.153 ± 0.050	**[A]p=0.008**
15	4.617 ± 0.076	1.410 ± 0.036	0.537 ± 0.006	**0.430 ± 0.010[A]**	**5.193 ± 0.090[A]**	5.033 ± 0.058	4.410 ± 0.017	**[A]p=0.009**

CO_2 formation by *S. cerevisiae* started on the second day after inoculation and for *M. pulcherrima* on the seventh day. However, gas production in the mixed populations exceeded *S. cerevisiae* on the fifth day. The notable increase in gas production in mixed populations was not statistically significant compared to *S. cerevisiae* (p>0.05, K-W test).

Figure S2. Box and whisker plots showing mean CO_2 formation after 7- and 15-day fermentation.

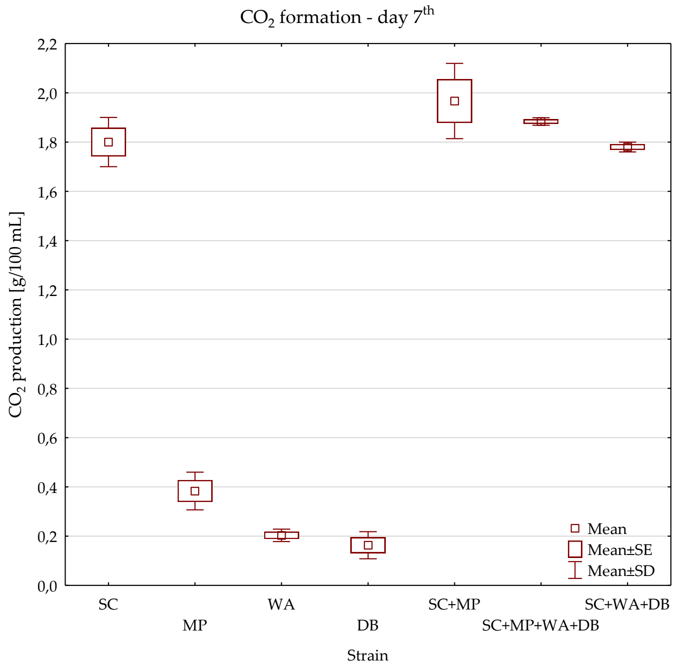

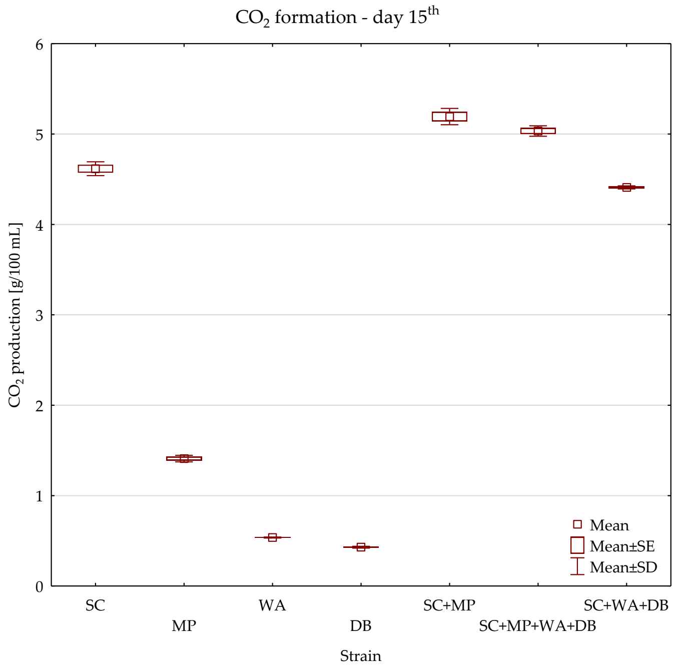

Figure S3. Box and whisker plots showing glucose and fructose content in monocultures and mixed populations.

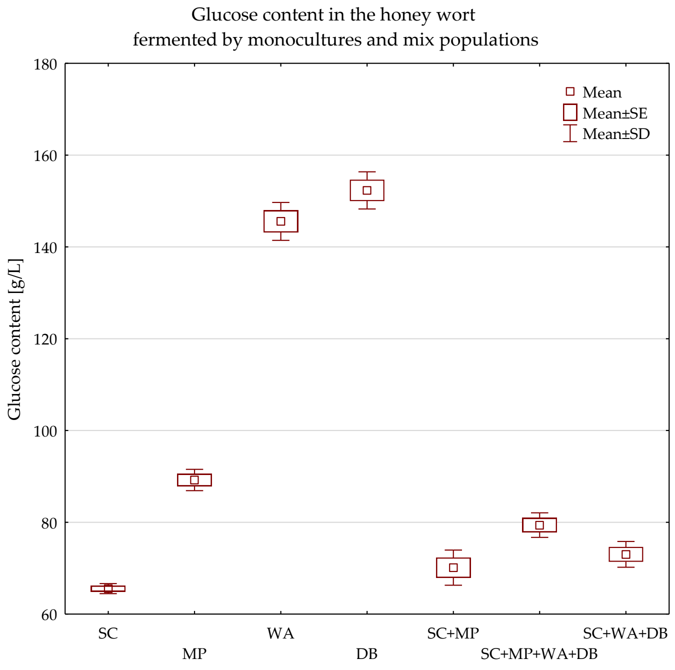

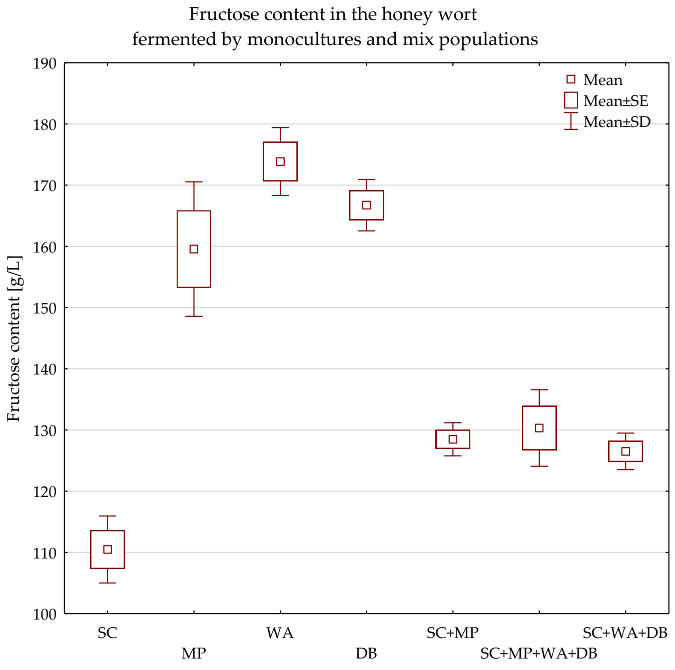

Figure S4. Box and whisker plots showing the compound content (glycerol, acetic acid, methanol, ethanol) in fermented honey beverages produced by tested yeast strains as monocultures and mixed populations.

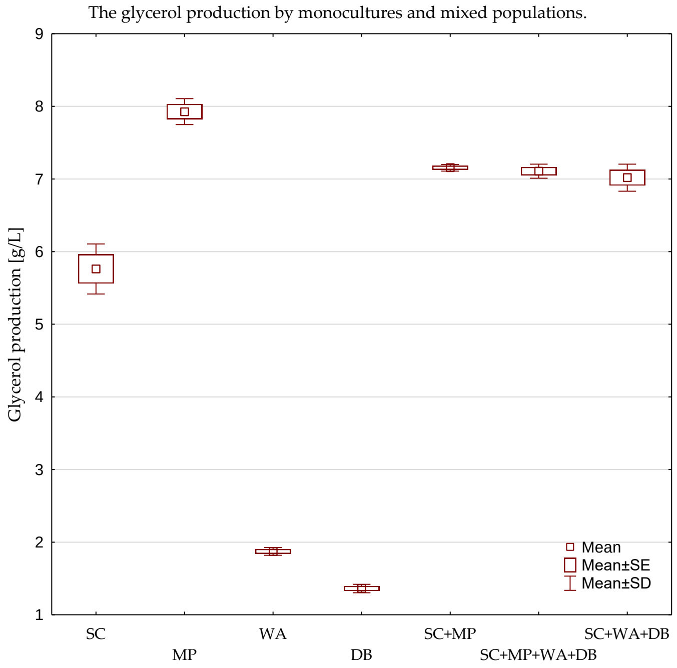

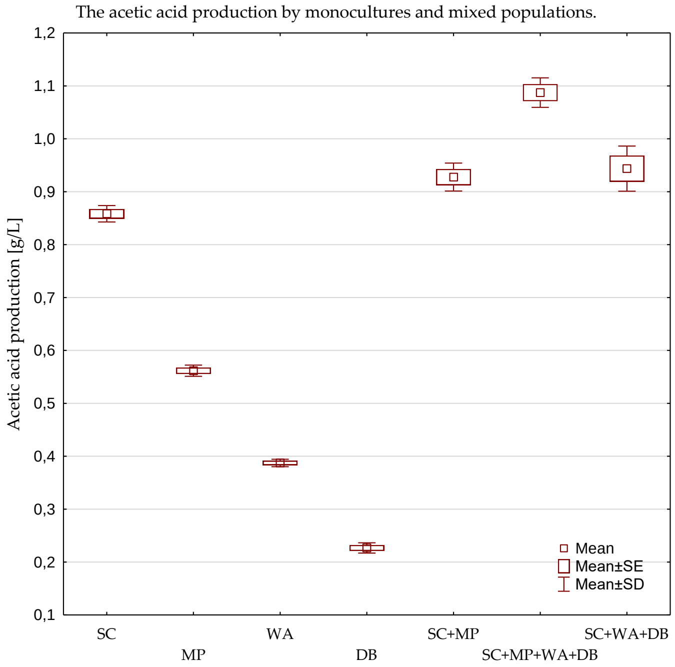

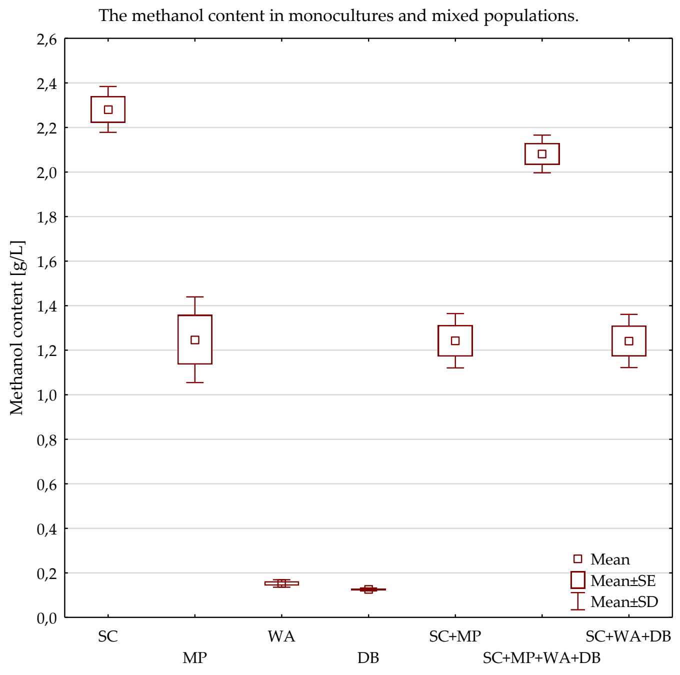
The methanol content in monocultures and mixed populations.
Methanol content [g/L]
2,6
2,4
2,2
2,0
1,8
1,6
1,4
1,2
1,0
0,8
0,6
0,4
0,2
0,0
Mean
Mean±SE
Mean±SD
SC
MP
WA
DB
SC+MP
SC+MP+WA+DB
SC+WA+DB

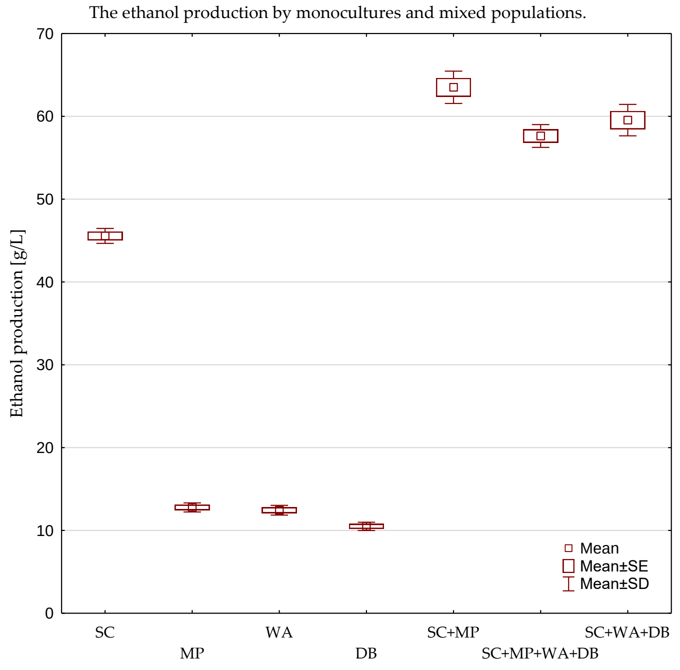
The ethanol production by monocultures and mixed populations.
Ethanol production [g/L]
70
60
50
40
30
20
10
0
Mean
Mean±SE
Mean±SD
SC
MP
WA
DB
SC+MP
SC+MP+WA+DB
SC+WA+DB

Table S3. Volatilomes [mg/L] of honey wort fermented by monocultures and mixed populations (data are presented as mean ± SD). The control sample is the honey wort before fermentation.

IUPAC NAME	Yeast strains							
	SC	MP	DB	WA	SC+MP	SC+MP+ DB+WA	SC+DB+ WA	Control sample
Acetaldehyde	75.513±2.384	138.881±3.866	3.535±0.101	21.902±0.703	47.128±1.862	35.761±1.074	38.806±1.206	nd
Propanal	nd	nd	0.039±0.001	0.041±0.001	0.182±0.007	0.046±0.001	nd	0.081±0.002
Ethyl formate	0.777±0.025	0.136±0.004	0.659±0.019	0.054±0.002	nd	0.369±0.011	0.480±0.015	nd
Propan-1-ol	2.129±0.067	3.050±0.085	nd	2.090±0.067	2.178±0.086	2.020±0.061	2.378±0.074	nd
Ethyl acetate	20.657±0.652	32.536±0.906	8.219±0.234	83.121±2.667	23.514±0.929	72.141±2.166	48.486±1.507	0.034±0.001
2-methylpropan-1-ol	15.843±0.500	25.039±0.697	17.734±0.505	2.296±0.074	nd	18.769±0.563	15.435±0.480	nd
Ethyl propanoate	0.012±0.001	0.072±0.002	nd	nd	nd	nd	0.066±0.002	nd
1,1-diethoxyethane	0.565±0.018	0.816±0.023	nd	nd	0.376±0.015	0.227±0.007	0.280±0.009	nd
3-methylbutan-1-ol	52.439±1.655	48.462±1.349	16.314±0.465	7.323±0.235	56.083±2.216	61.214±1.838	65.180±2.026	nd
2-methylbutan-1-ol	14.449±0.456	12.35±0.344	3.968±0.113	2.693±0.086	16.228±0.641	16.210±0.487	17.324±0.539	nd
Ethyl 2-methylpropanoate	nd	nd	nd	nd	nd	0.007±0.000	0.003±0.000	nd
2-methylpropyl acetate	nd	nd	0.004±0.000	nd	nd	0.006±0.000	0.004±0.000	nd
Ethyl butanoate	nd	nd	nd	nd	nd	0.006±0.000	0.002±0.000	nd
3-methylbutyl acetate	0.012±0.000	0.010±0.000	nd	nd	0.005±0.000	0.063±0.002	0.064±0.002	nd
Ethyl hexanoate	0.003±0.000	0.004±0.000	nd	nd	nd	0.011±0.000	0.005±0.000	nd
Ethyl octanoate	nd	nd	nd	nd	nd	0.010±0.000	0.003±0.000	nd
nd – not detected								
